# Evaluation of a pixelated large format CMOS sensor for x‐ray microbeam radiotherapy

**DOI:** 10.1002/mp.13971

**Published:** 2020-01-06

**Authors:** Samuel Flynn, Tony Price, Philip P. Allport, Ileana Silvestre Patallo, Russell Thomas, Anna Subiel, Stefan Bartzsch, Franziska Treibel, Mabroor Ahmed, Jon Jacobs‐Headspith, Tim Edwards, Isaac Jones, Dan Cathie, Nicola Guerrini, Iain Sedgwick

**Affiliations:** ^1^ School of Physics and Astronomy University of Birmingham Birmingham B15 2TT UK; ^2^ Medical Physics Department National Physical Laboratory Teddington TW11 0LW UK; ^3^ UCL Cancer Institute University College London London WC1E 6AG UK; ^4^ Helmholtz Centre Munich Institute for Radiation Medicine Munich 85764 Germany; ^5^ School of Medicine Klinikum rechts der Isar Department of Radiation Oncology Technical University of Munich Munich 80333 Germany; ^6^ vivaMOS Ltd Southampton SO16 7NP UK; ^7^ Rutherford Appleton Laboratory Didcot OX11 0QX UK

**Keywords:** CMOS detectors, compact microbeam sources, dosimetry, microbeam radiation therapy

## Abstract

**Purpose:**

Current techniques and procedures for dosimetry in microbeams typically rely on radiochromic film or small volume ionization chambers for validation and quality assurance in 2D and 1D, respectively. Whilst well characterized for clinical and preclinical radiotherapy, these methods are noninstantaneous and do not provide real time profile information. The objective of this work is to determine the suitability of the newly developed vM1212 detector, a pixelated CMOS (complementary metal‐oxide‐semiconductor) imaging sensor, for *in situ* and *in vivo* verification of x‐ray microbeams.

**Methods:**

Experiments were carried out on the vM1212 detector using a 220 kVp small animal radiation research platform (SARRP) at the Helmholtz Centre Munich. A 3 x 3 cm^2^ square piece of EBT3 film was placed on top of a marked nonfibrous card overlaying the sensitive silicon of the sensor. One centimeter of water equivalent bolus material was placed on top of the film for build‐up. The response of the detector was compared to an Epson Expression 10000XL flatbed scanner using FilmQA Pro with triple channel dosimetry. This was also compared to a separate exposure using 450 µm of silicon as a surrogate for the detector and a Zeiss Axio Imager 2 microscope using an optical microscopy method of dosimetry. Microbeam collimator slits with range of nominal widths of 25, 50, 75, and 100 µm were used to compare beam profiles and determine sensitivity of the detector and both film measurements to different microbeams.

**Results:**

The detector was able to measure peak and valley profiles in real‐time, a significant reduction from the 24 hr self‐development required by the EBT3 film. Observed full width at half maximum (FWHM) values were larger than the nominal slit widths, ranging from 130 to 190 µm due to divergence. Agreement between the methods was found for peak‐to‐valley dose ratio (PVDR), peak to peak separation and FWHM, but a difference in relative intensity of the microbeams was observed between the detectors.

**Conclusions:**

The investigation demonstrated that pixelated CMOS sensors could be applied to microbeam radiotherapy for real‐time dosimetry in the future, however the relatively large pixel pitch of the vM1212 detector limit the immediate application of the results.

## 1 Introduction

### 1.1 Microbeam radiotherapy

Microbeam radiotherapy (MRT) is a novel type of spatially fractionated therapy which is defined by narrow beams of radiation (typically <100 µm) that can selectively irradiate portions of the target volume.^1^ To cover the entire target volume, microbeams are delivered in a grid pattern in which multiple quasi‐parallel rectangular beams, with typical centre‐to‐centre distances of 200–400 µm. Crucially the entire target volume is not irradiated uniformly, with regions of very high dose microbeam "peaks" separated by very low dose valleys.

Preclinical studies have indicated that this dose pattern has a greater efficacy than that of a single uniform field.^2^ Whilst the exact mechanism for preferential effect tumor is not fully understood and is likely a combination of effects. Possible mechanisms under investigation are preferential damage to vascular tissue in tumors,^3^, ^4^, ^5^ and radiation‐induced bystander and abscopal effects.^6^, ^7^

### 1.2 Current verification methods

The very small size and high dose gradients of microbeams present a significant challenge to most standard detectors. That combined with high dose rates at synchrotrons adds to the complexity when working towards accurate dosimetry for microbeam radiotherapy.

Stereotactic radiotherapy treatments (with radiation fields sizes typically between 0.4 and 30 mm^8^) have strict requirements on the geometrical and dosimetric accuracy from dose calculations to delivery of ± 5% (k = 2).^9^ Microbeam irradiations are a step forward in terms of complexity and at present there is no dosimetry protocol or recommendations for dosimetry of irradiations with such beam configurations.^10^

Much of the ongoing research in the community is dedicated to optimizing irradiation configurations in order to obtain the best therapeutic outcomes, with peak to peak distance,^11^ full‐width at half maximum (FWHM)^12^ and the peak‐to‐valley dose ratio (PVDR)^13^, ^14^ being of particular interest.

Due to the very small scales involved in microbeam radiotherapy, conventional radiotherapy equipment for beam profile acquisition (like small volume ionization chambers) are unable to resolve the individual microbeam peaks^9^^)^. Scanning other types of small volume detectors through a microbeam peak has been previously performed with success by using a MOSFET dosimeter^15^, ^16^ or with a commercial PTW (Physikalisch‐Technische Werkstätten GmbH, Freiburg, Germany) microdiamond detector,^17^ with resolutions of 1 µm.^18^ This method has shown good agreement with radiochromic film,^19^ however the measurements are acquired point by point and therefore the shape of the profiles are not shown instantaneously which limits its use for *in vivo* dosimetry or *in situ* verification. The same applies to the use of scintillating fibers, as shown by Archer *et al.* 20

Various groups have developed silicon strip detectors capable of quantifying parameters of the microbeam field.^21^, ^22^, ^23^ Whilst hybrid strip detectors (with separate sensor and readout) can offer greater resistance to radiation than monolithic pixelated detectors, strip detectors do not provide detailed information about the 2D profile of the radiation field and, therefore, will be more sensitive to angular misalignment.

A method of obtaining 2D relative dose distributions of microbeams was developed by Bartzsch *et al*.^24^ using optical microscopy and EBT3 films,^25^ which when using a microscope is technically capable of spatial resolutions better than 1 µm. Due to film grain inhomogeneities this is reduced to 5 µm in practice. This method builds on existing techniques for film dosimetry. Radiochromic films have a relatively large dose range (0.1 cGy–10 Gy for EBT3^26^), however the analysis process is slow, requiring a minimum of 24 hr for self‐development post‐irradiation.^27^ At lower dose levels (less than 0.1 Gy^28^, ^29^) noise becomes more significant. This typically necessitates two separate sets of irradiations for the same set of microbeams, in order to be able to increase the accuracy of the assessment of the dose distribution in the regions with lower dose range (valleys) without saturating the high dose region of the microbeam peaks.

This investigation was carried out to evaluate the suitability of the newly developed vM1212 detector for its use in the analysis of preclinical radiotherapy microbeams, using the custom built multi‐slit collimator at the Helmholtz Zentrum München, Germany. The objective was to quantify microbeam parameters and to compare the results of the analysis of the same deliveries to EBT3 films, using the optical microscopy method.^24^

## 2 Materials And Methods

### 2.1 vM1212 pixelated detector

The vM1212 pixelated detector is a large format CMOS (complementary metal–oxide–semiconductor) imaging sensor with 50 µm pixel pitch originally designed for medical and scientific x‐ray imaging by the CMOS Sensor Design Group at the Rutherford Appleton Laboratory^30^ and is now licensed and manufactured into a full detector assembly by vivaMOS Ltd. The active area of the vM1212 detector is approximately 6 × 6 cm^2^ (1204 × 1248 pixels) and is sufficiently large to capture the entire radiation field of the microbeam multislit collimator in a single instance.

The small pixel pitch and predicted resistance to damage caused by high levels of ionizing radiation justified a proof of principle investigation to determine the response of the detector to microbeam radiation.

### 2.2 Methodology

A SARRP (Small Animal Radiation Research Platform) x‐ray irradiator at the Helmholtz Zentrum München was used for this investigation. The irradiation parameters were set to 220 kVp (0.67 mm Cu HVL); 2.8 mA; and fine focus (effective beam source size of 0.4 mm^31^).

**Figure 1:**
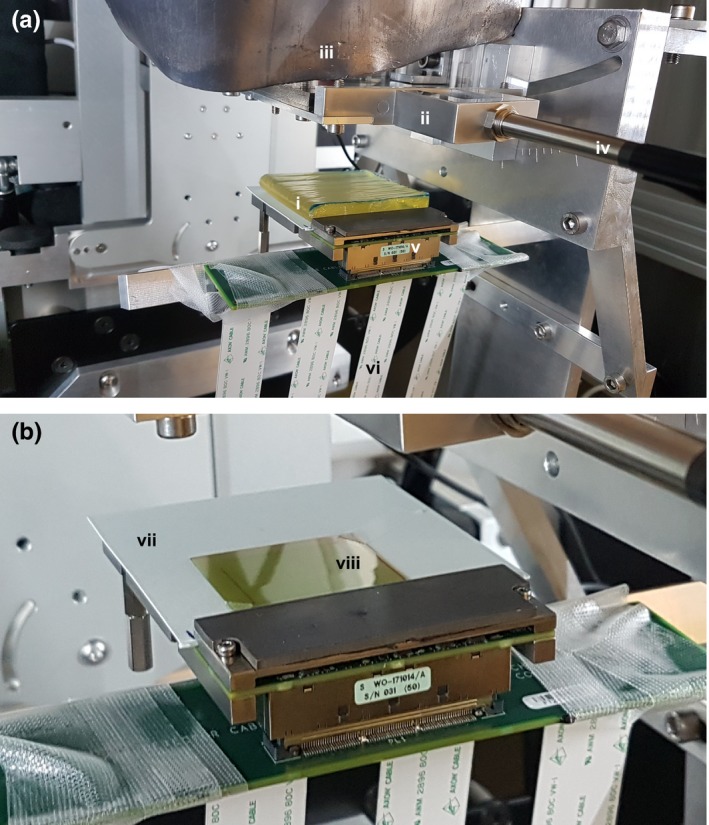
Experimental set up: (a) vM1212 detector with 1 cm of water equivalent build‐up, (b) vM1212 detector with aligned EBT3 Film. (i) Lead shield to protect collimator electronics; (ii) Microbeam collimator; (iii) 1 cm of water equivalent bolus; (iv) Cable for microbeam collimator; (v) vM1212 detector; (vi) Ribbon cables for vM1212 detector; (vii) Nonfibrous card with alignment points; (viii) 3 x 3 cm^2^ square of EBT3 film. [Color figure can be viewed at http://wileyonlinelibrary.com]

The tungsten microbeam multislit collimator consisted of three levels of fifty one 100‐µm slits (7 mm total thickness), with a slit‐to‐slit separation of 400 µm. The first and third levels are in a fixed alignment, whilst the second central level is controlled by two motorized translation stages. When fully open, the transmission gap is 100 µm, but the finest step resolution of the piezoelectric pistons enables variable slit widths between 0 and 100 µm to be investigated to an accuracy of 0.5 µm. The collimator was mounted at a distance of 21.2 cm from the source, with additional lead shielding to prevent radiation damage to the electronics as shown in Fig. 1(a).

In order to obtain robust and safe positioning, the vM1212 detector had to be mounted at a source to surface distance (SSD) of 29 cm, 6.8 cm from the surface of the microbeam collimator. To achieve conditions similar to the ones used for small animal irradiations a 1 cm slab of tissue‐equivalent flexible bolus material with density of 1.03 g/cm^32^ (trimmed to 7 × 7 cm^2^) was placed on top of the EBT3 film. The vM1212 detector was used without scintillating material to maximize the potential spatial resolution. To enable a direct comparison between the EBT3 film and the vM1212 detector, EBT3 film pieces were placed on top of the active area of the sensor, separated by a thin layer of a nonfibrous card which had been marked for repeatable alignment [Fig. 1(b)].

The EBT3 films irradiated simultaneously to the vM1212 detector were scanned using an Epson Expression 10000XL flatbed scanner (1400 dpi) and calibrated using FilmQA Pro with triple channel dosimetry.^33^, ^34^ Due to time constrains during the experiment, it was not possible to irradiate a second set of films for their analysis with optical microscopy. Those irradiations were performed in an independent experiment following the same irradiation conditions: source‐surface distance, same bolus material and nonfibrous card, but using 450 µm of silicon simulating the thickness of the detector. This second set of films was scanned using a ZEISS Axio Imager 2 optical microscope^35^ on 5X magnification for a pixel resolution of 1.29 µm.

Prior to the film irradiations, the output (Gy/min) was measured in reference conditions for SARRP absolute calibration. Measurements were performed with the SARRP open field at Source Surface Distance (SSD) of 33 cm and at 2 cm depth in WT1 water equivalent slab phantom, with 3 cm of backscatter material. Two independent measurements of the SARRP output were performed, one with the local dosimetry system (PTW 30010 ionization chamber), traceable to the PTW‐Freiburg SSDL Calibration Laboratory and with a National Physical Laboratory (NPL) secondary standard system (PTW 30012 ionization chamber), traceable to the NPL primary standard for medium energy x‐rays. Both ionization chambers used a local PTW Unidos TW1001 electrometer for dosimetry. Following output measurements and in order to obtain a calibration curve, a set of nine films were irradiated in the same reference conditions, with doses ranging from 0 to 14 Gy.

For consistency throughout the investigation, the same integration time, 28 ms, was always used on the vM1212 detector. This ensured that all the performed measurements were all in the linear response region for the pixels and prevented saturation of the detector. The results obtained using the vM1212 detector were corrected by averaging over a number of frames to reduce noise, subtracting a dark image to account for dark current in the pixels and calibrating the pixel response values against measurements with the NPL ionization chamber under the same conditions.

Direct comparison between the EBT3 films and the different acquisitions with vM1212 detector were carried out for 25, 50, 75, and 100 µm slit widths. All the slits were irradiated with 240 s of exposure with the exception of the 25 µm slit width which was irradiated with 480 s, to increase the dose and therefore to reduce the level of noise for the films measurements in such narrow beams.

Finally, to understand the difference in spatial response between the vM1212 detector and the two methods of EBT3 film scanning, the modulation transfer function (MTF) was measured for each. The modulation transfer function of the vM1212 detector was measured following BS EN 62220‐1‐3:2008^36^ and using the COQ analysis software written by Donini *et al.* 37. The MTF of the Epson Expression 10000XL scanner at 1400 dpi scanning resolution was measured using a sharp flat edge positioned over a piece of unexposed EBT3 film at an angle of 4°. Again using the COQ analysis software, the edge spread function was calculated allowing the modulation transfer function to be determined. The MTF of the Zeiss Axio Imager 2 was measured with the Xradia resolution sample (provided by Zeiss), which contained a pattern of lines with known width and separation. The largest line width on this pattern was 32 µm (period = 64 µm), and as such the smallest resolution measurable with this resolution sample was 15.6 line pairs/mm (1/0.064 mm).

## 3 Results

### 3.1 Profile measurements

**Figure 2:**
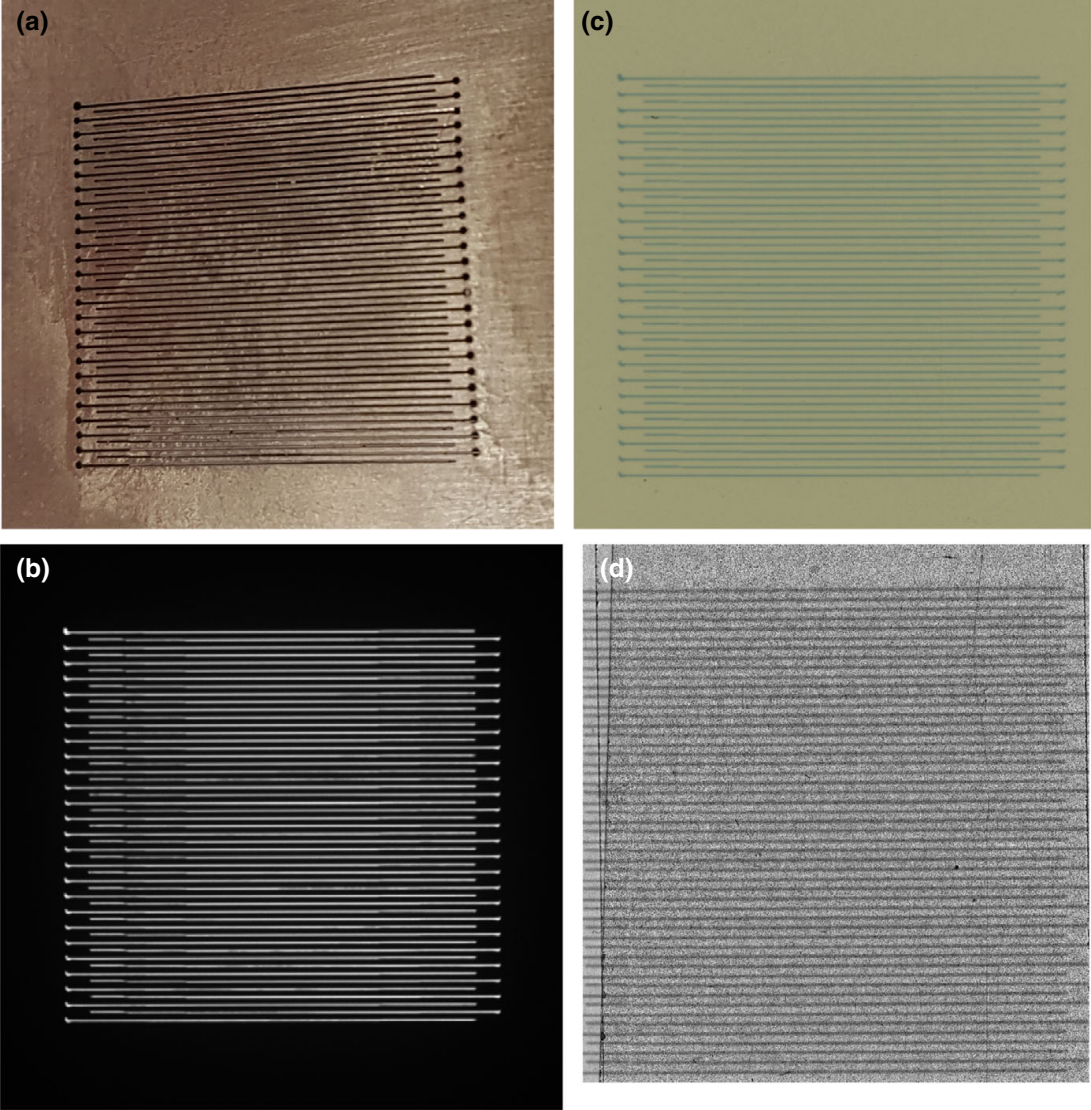
(a) Photograph of microbeam collimator slits. (b) vM1212 detector image (cropped). (c) Scan of exposed EBT3 film using the Epson Expression 10000XL scanner (100 µm slit width). (d) Scan of exposed EBT3 film using Zeiss Axio Imager 2. [Color figure can be viewed at http://wileyonlinelibrary.com]

It was found that the vM1212 detector was able to capture the entire radiation field as defined by the collimator, as can be seen in Fig. 2(b). To create the microbeam collimator slits in tungsten, 0.3 mm diameter holes had to be drilled into the tungsten, allowing for wire erosion to mill out the 100 µm wide slits. This detail can be recognized on both detectors (film and vM1212 detector) and was used for alignment purposes. All profile comparisons presented are aligned relative to the central 26th peak. By comparing vertical profiles from the EBT3 film methods with vertical profiles taken using the vM1212 detector we were able to observe that the alternating pattern of peaks and valleys of the microbeam collimator are well correlated between the different detectors. The larger SSD required to mount the vM1212 detector and the maximum scanning size of the EBT3 film possible with the microscope reduced the number of peaks that could be recorded using this method to approximately 40 (reduced from 51 physical slits on the collimator).

**Figure 3:**
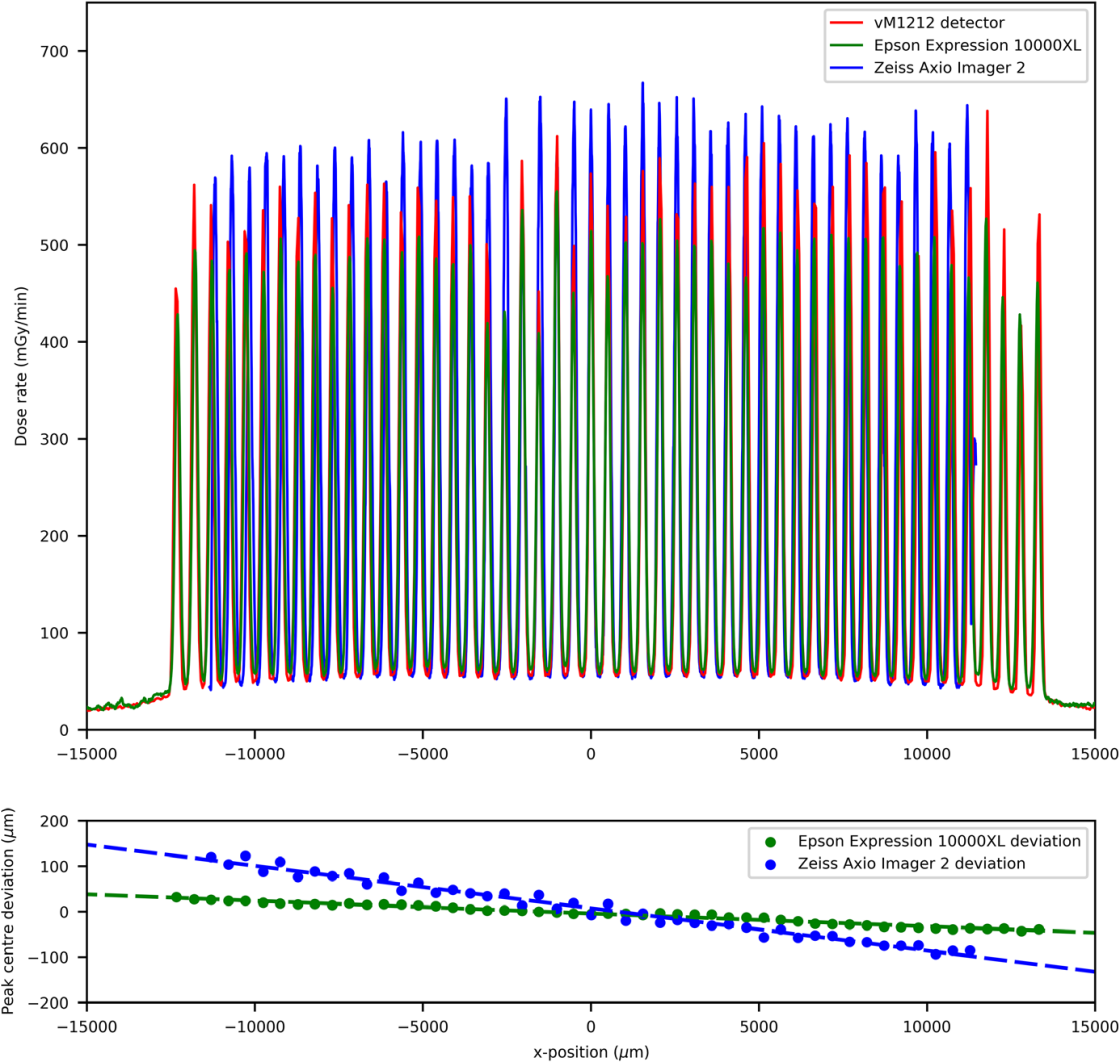
(a) 100 µm slit width profile comparison, (b) Microbeam peak deviation between the vM1212 detector and the two EBT3 film methods. [Color figure can be viewed at http://wileyonlinelibrary.com]

**Figure 4:**
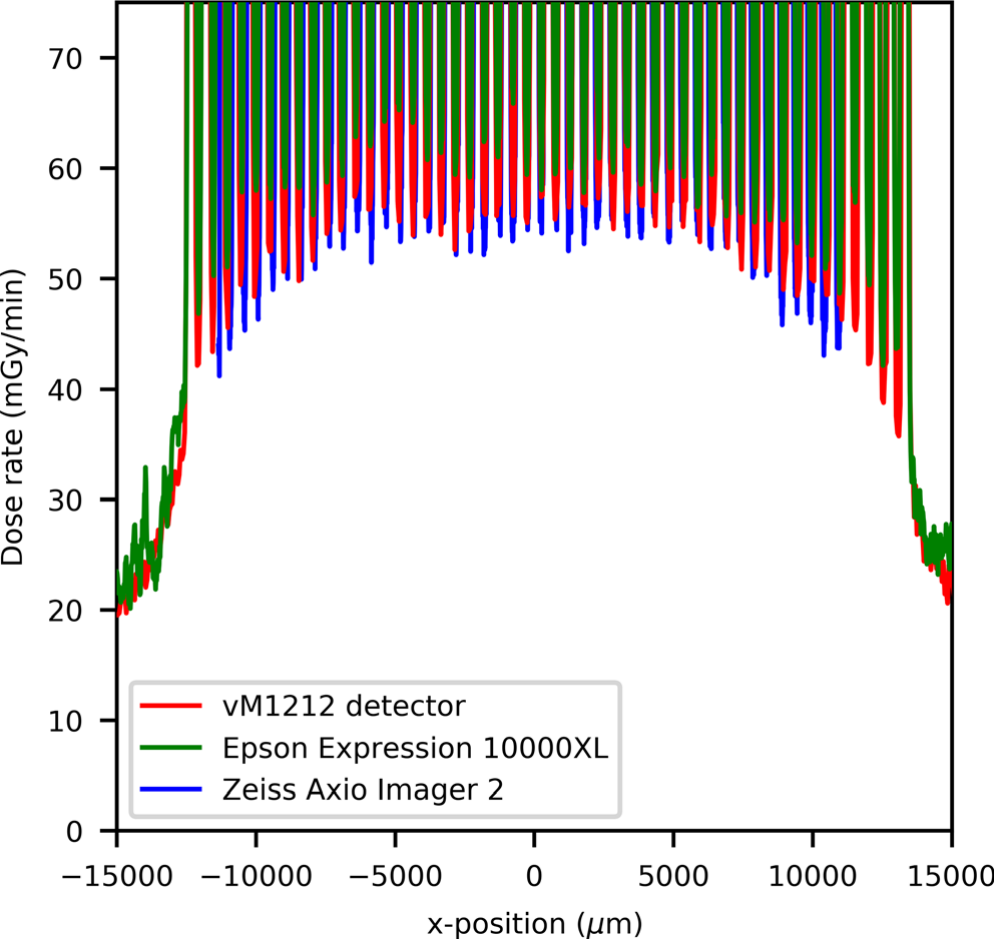
100 µm slit width valley profile comparison. [Color figure can be viewed at http://wileyonlinelibrary.com]

**Figure 5:**
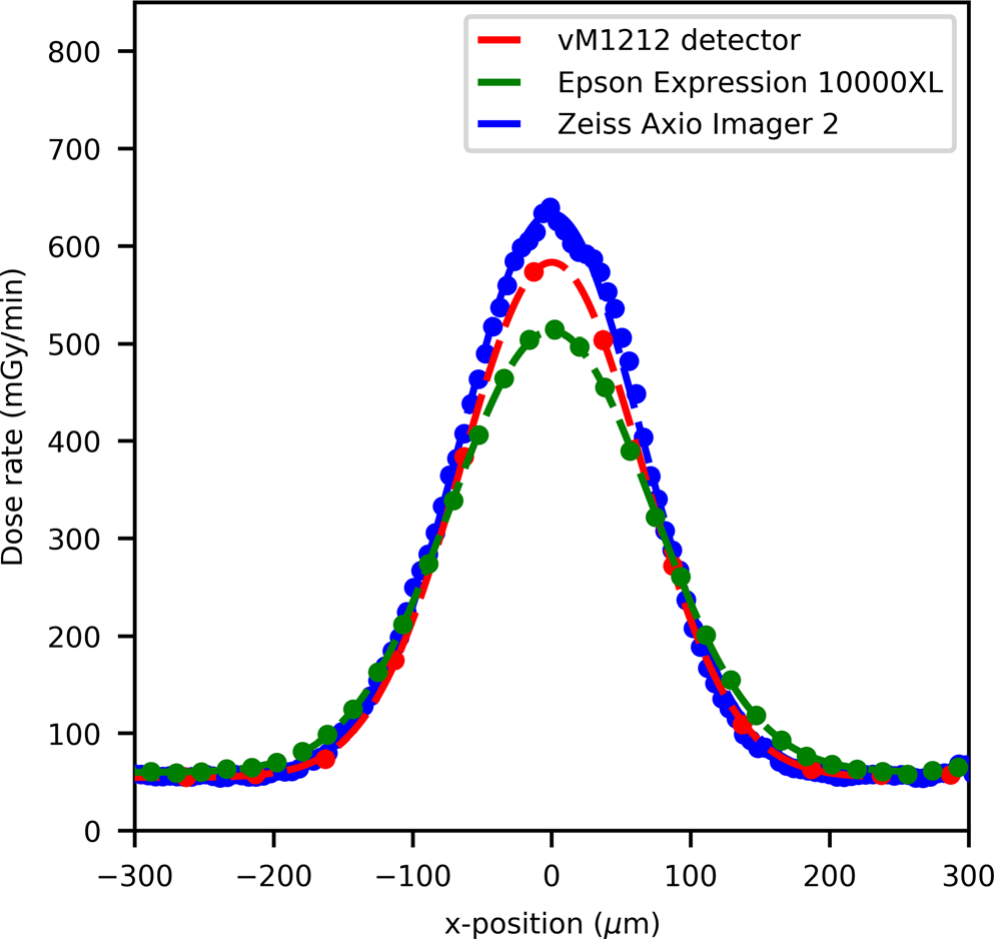
100 µm slit width profile comparison of the 26th central peak. [Color figure can be viewed at http://wileyonlinelibrary.com]

The 100 µm slit profiles’ comparison can be seen in Fig. 3(a), where an agreement in terms of alignment of the peaks between the three detector methods can be observed. The vM1212 detector and the Epson Expression 10000XL under respond in terms of peak dose by approximately 30%; however there is relatively good agreement of the location of the microbeam peak center values [Fig. 3(b)]. As shown in Fig. 4, relative to the Zeiss Axio Imager 2, the valley doses are over reported by the Epson Expression 10000XL (with scanning resolution at 1400 dpi) by approximately 25% (15 mGy/min), whilst the vM1212 detector over reports by less than 5% (5 mGy/min). The average deviation between corresponding peak centers for the vM1212 detector and the Epson Expression 10000XL measurement was 18.5 µm, whilst for the Zeiss Axio Imager 2 measurement was found to be 55.3 µm. As shown in Fig. 5 for the 26th central peak, the profile resolved on all three detector methods appears to be Gaussian.

**Figure 6:**
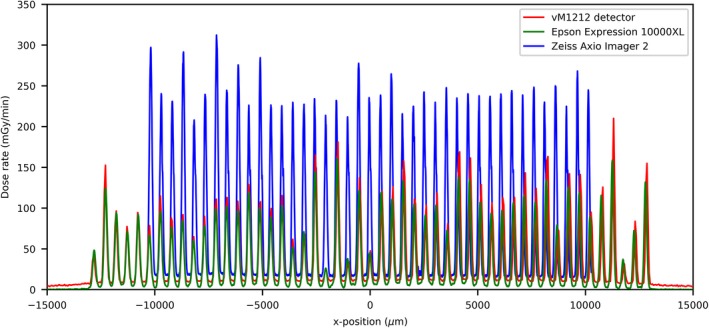
25 µm slit width profile comparison. [Color figure can be viewed at http://wileyonlinelibrary.com]

**Figure 7:**
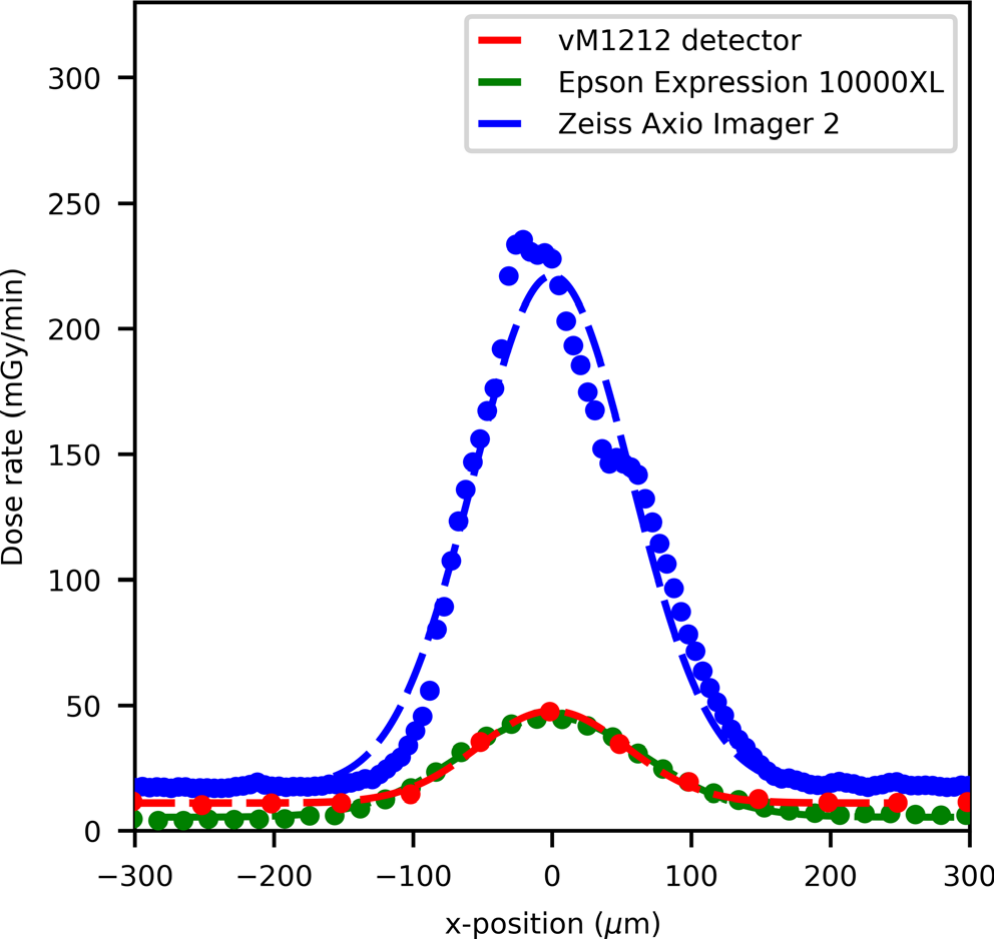
25 µm slit width peak profile comparison of the 26th central peak. [Color figure can be viewed at http://wileyonlinelibrary.com]

**Figure 8:**
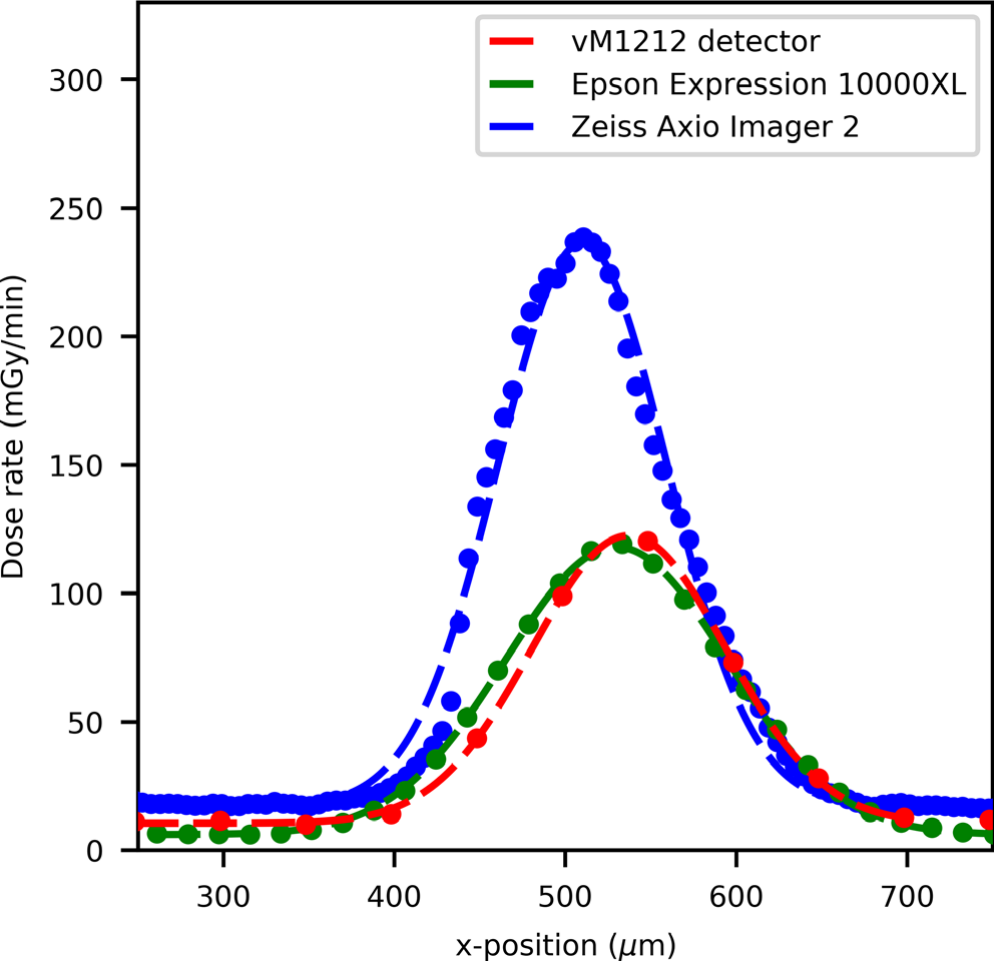
25 µm slit width peak profile comparison of the 27th peak. [Color figure can be viewed at http://wileyonlinelibrary.com]

For the 25 µm slit width comparison (Fig. 6) the agreement between the EBT3 films and the vM1212 detector becomes worse as there is a strong disagreement for dose rate values between the scan performed by the Zeiss Axio Imager 2 and the other methods. This deviation is likely due to spatial averaging within the vM1212 detector and the Epson Expression 10000XL, however it is also possible that this deviation was introduced by misalignment during the Zeiss Axio Imager 2 exposure as it was performed at a later date. The lower measured dose rate is not consistent across the microbeam profiles as shown for the central peak (Fig. 7), where the dose rate measured by the vM1212 detector and Epson Expression 10000XL EBT3 film is approximately 20% of the dose rate measured by the Zeiss Axio Imager 2. For the Epson Expression 10000XL and the vM1212, the dose rate measured for the 27th peak (Fig. 8) is better but still measures only 40% relative to the Zeiss Axio Imager 2. Valley profiles for the 25 µm slit measured all of the detectors are again inconsistent, with approximate differences relative to the Zeiss Axio Imager 2 of 40% and 20% for the vM1212 detector and Epson Expression 10000XL, respectively. This peak specific under response not observed in the Zeiss Axio Imager 2 measurement is suspected to be due to a combination of manufacturing tolerances on the machined microbeam slits and repeatability issues of the microbeam setup.

Figures 7, 8 and 8 show a profile comparisons with a Gaussian fit applied between the three detectors for the 26th (central) and 27th peak, respectively. A stitching artifact between the high dose valley irradiation and the low dose peak measurement can been seen in Fig. 8 in the Zeiss Axio Imager 2 dose profile at approximately 50 µm. The centers of the 27th microbeam peak (relative to the 26th central peak) can be calculated to be 550, 514, and 488 µm for the vM1212 detector, Epson Expression 10000XL and Zeiss Axio Imager 2 respectively.

The peak to peak separation could be measured across the three detection methods for all measured slit widths, as shown in Table 1. It can be shown that the three methods agree within the uncertainties calculated. Using the inverse square law and the differences between the measured peak to peak separations, it can be estimated that the EBT3 films for the Epson Expression 10000XL and Zeiss Axio Imager 2 measurements were positioned 0.5 ± 0.2 mm and 2.4 ± 0.2 mm closer respectively to the x‐ray source than the vM1212 detector measurement. As the measurements for the Epson Expression 10000XL were taken concurrently with the vM1212 detector, this difference can be attributed to the thickness the nonfibrous card which was independently measured with a digital caliper to be 0.53 ± 0.01 µm. The 2.4 mm deviation of the Zeiss Axio Imager 2 measurement is likely due to setup misalignment.

**Table 1:** Measured peak to peak separation as measured on the three detectors. Statistical uncertainty corresponds to one standard deviation.

Nominal slit width (µm)	Measured peak to peak separation (µm)		
	vM1212 detector	Epson Expression 10000XL	Zeiss Axio Imager 2
25	513.4 ± 13.9	512.0 ± 11.3	508.3 ± 9.9
50	512.9 ± 10.1	511.7 ± 9.7	508.9 ± 9.1
75	512.6 ± 9.2	511.9 ± 10.1	508.3 ± 8.6
100	512.4 ± 9.5	511.8 ± 9.6	508.5 ± 9.8

**Figure 9:**
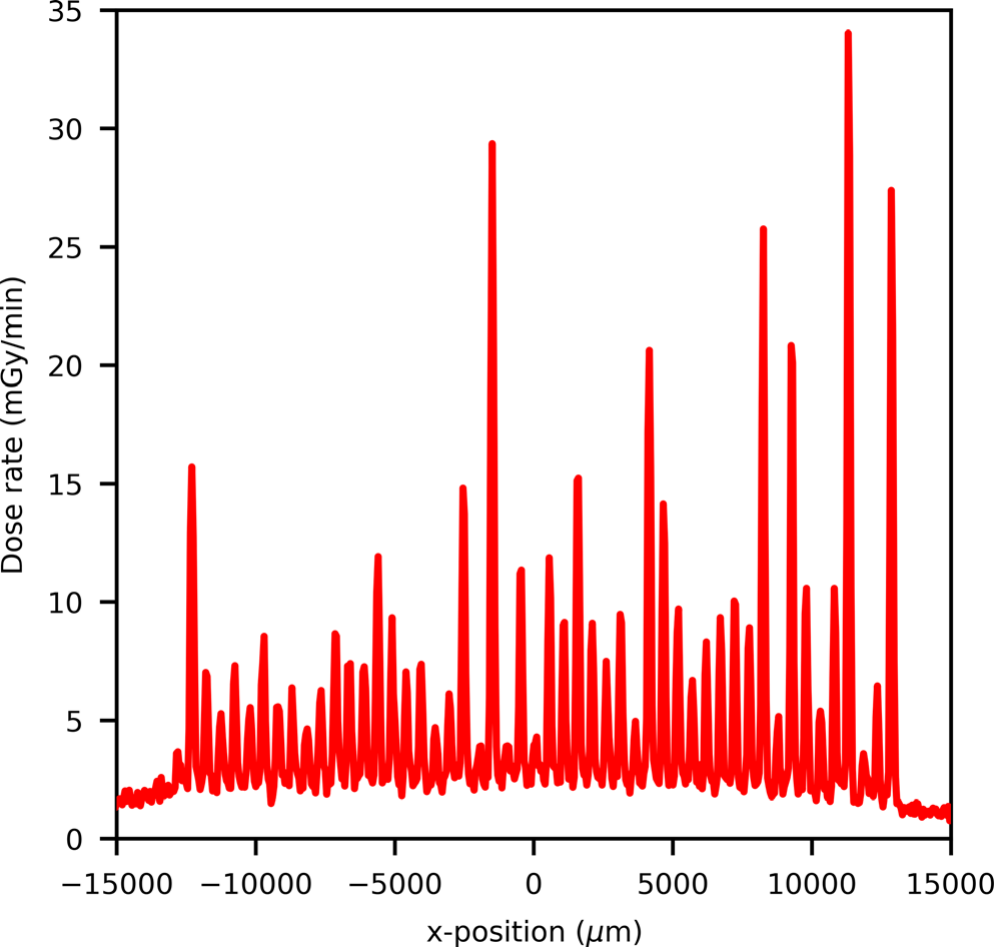
Radiation leakage through the collimator at 0 µm slit width as measured by the vM1212 detector. [Color figure can be viewed at http://wileyonlinelibrary.com]

It was also found that the vM1212 detector was still able to detect and identify each of the 51 peaks when the microbeam collimator is fully closed (set to 0 µm slit width) (Fig. 9). Profiles resulting from this leakage are used in Sections 3.B FWHM measurements and 3.C Peak and Valley Measurements.

**Figure 10:**
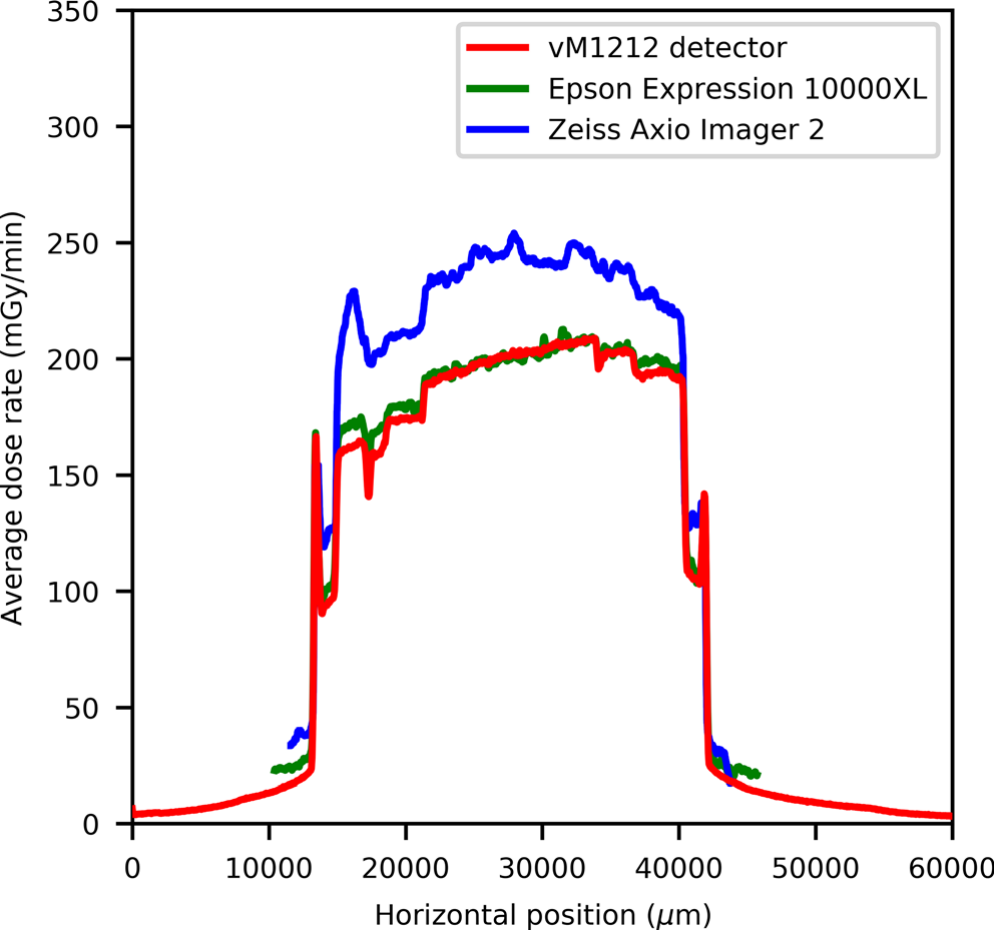
Horizontal profile of the 100 µm slit width. [Color figure can be viewed at http://wileyonlinelibrary.com]

Using the vM1212 detector it is possible to take real time horizontal profiles of the microbeam collimator. A comparison between the methods averaged across all recorded peaks for the 100 µm slit width can be seen in Fig. 10, which again shows the approximately 30% under response of the vM1212 detector and Epson Expression 10000XL measurements relative to the Zeiss Axio Imager 2 measurement. The sharp vertical peaks at 13,000 and 41,000 µm are due to the 0.3 mm diameter holes seen in Fig. 2. It can be seen in all three methods that the radiation intensity does not follow a smooth profile across the collimator as one might expect, although it is beyond the scope of this paper to discuss any therapeutic impact this may have.

### 3.2 FWHM measurements

**Figure 11:**
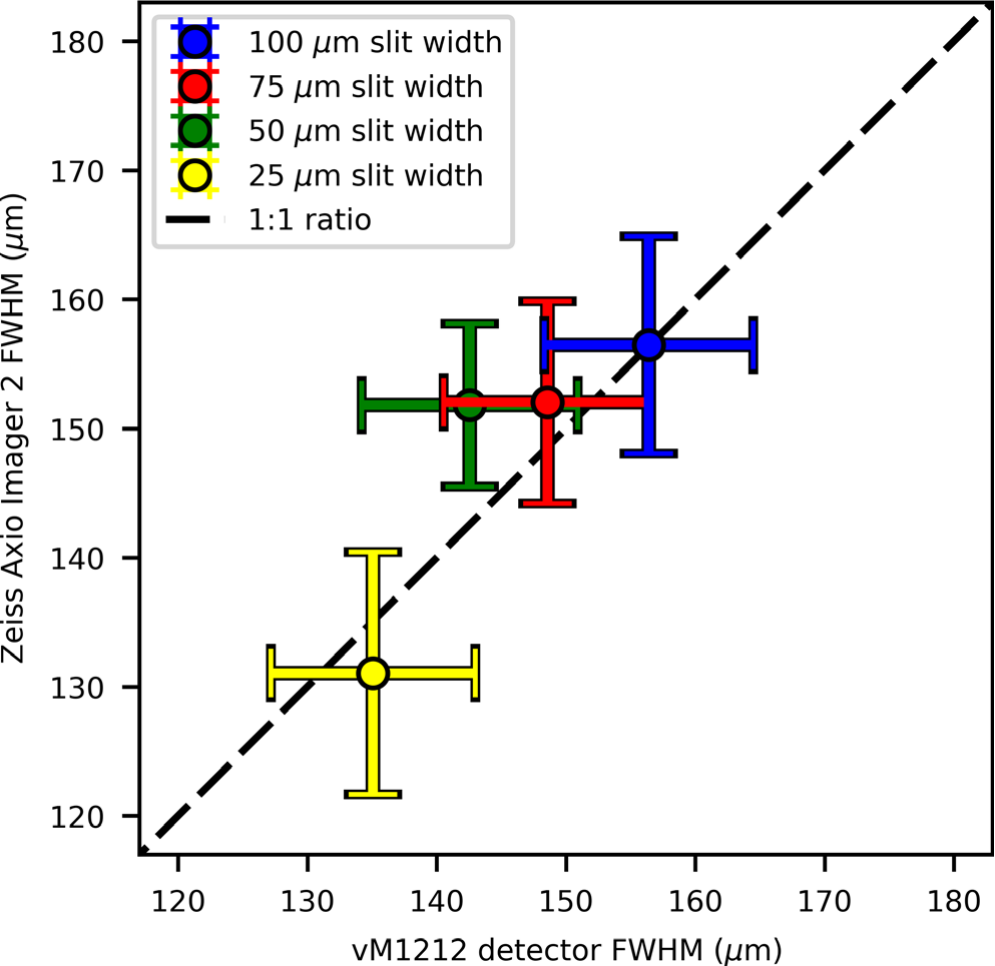
FWHM comparison between Zeiss Axio Imager 2 and the vM1212 detector. A 1:1 ratio has been added to guide the eye. [Color figure can be viewed at http://wileyonlinelibrary.com]

An averaged FWHM comparison between the Zeiss Axio Imager 2 and the vM1212 detector for each of the slits can be seen in Fig. 11. The error bars shown represent one standard deviation of uncertainty for the microbeam peaks.

**Figure 12:**
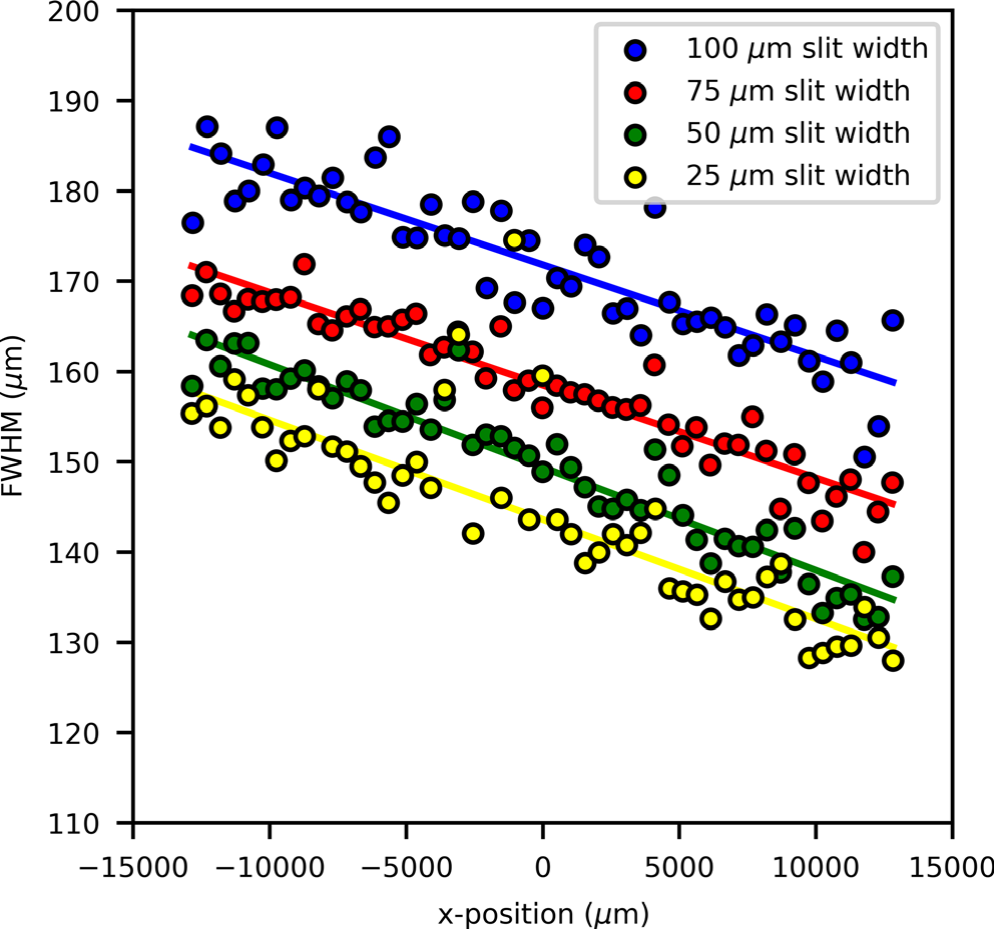
FWHM trend as measured by the vM1212 detector. [Color figure can be viewed at http://wileyonlinelibrary.com]

A linear relationship between the FWHMs is observed; however, there is a large deviation between FWHMs within a measurement. This can be attributed to a significant trend in the FWHM as a function of vertical position that was undetectable at the time of the experiment that can be seen in both the vM1212 detector results (Fig. 12) and the analyzed EBT3 films (not shown). This is most probably due to the angle of the beam after it is produced at the tungsten target, within the x‐ray tube, known as heel effect. This effect would have become more dominant due to the larger SSD and was not observed on past measurements using the microbeam collimator.

Such a difference in beam FWHM across the beam profile would have had a significant impact on patient outcome, as described by Serduc *et al.* 12. For *in vivo* verification this would have been impossible to diagnose in real time with EBT3 films, due to the minimum 24 hr time required for film self‐development. This highlights a potential application of the vM1212 detector for real time imaging of microbeams.

**Figure 13:**
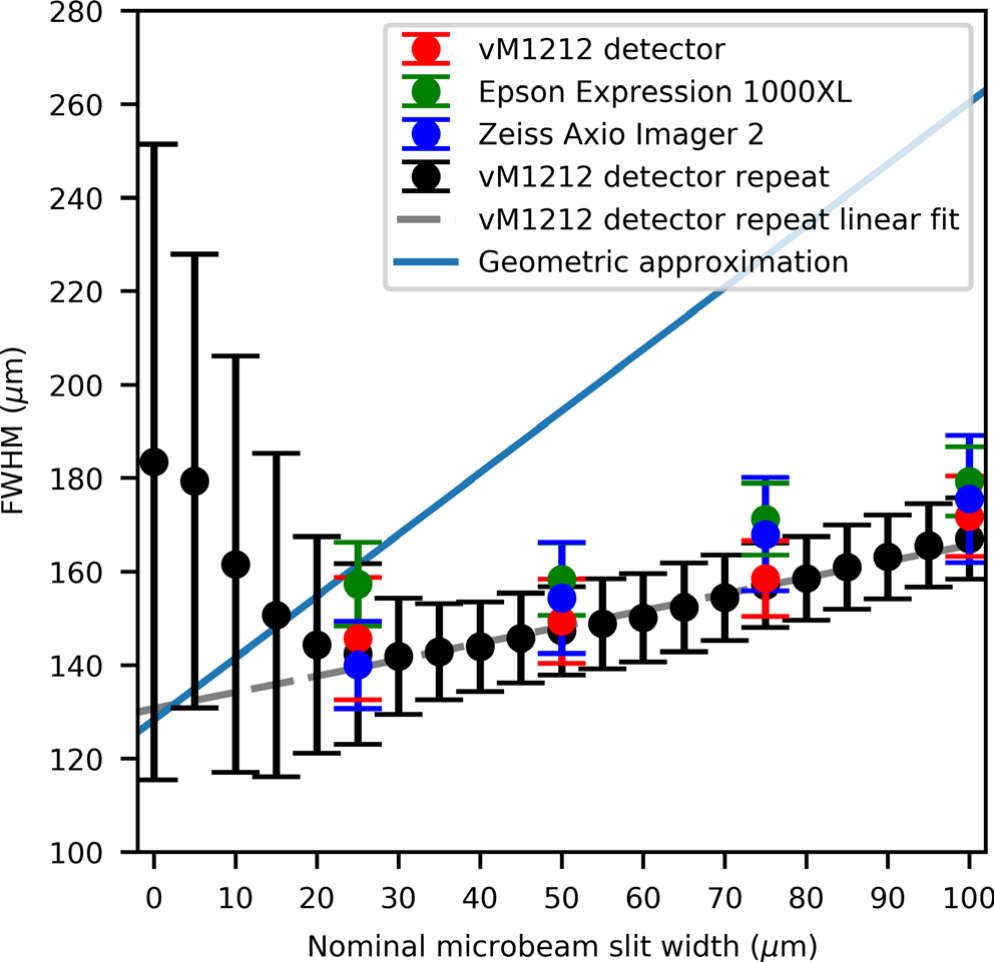
Comparing the microbeam slit width to observed FWHM. [Color figure can be viewed at http://wileyonlinelibrary.com]

A comparison of microbeam nominal slit width to the measured FWHM can be seen in Fig. 13. As the vM1212 detector could take multiple readings with minimal dead time between them, a repeat set of measurements was performed to calculate the FWHM of the microbeams. Each time the slit width was increased by 5 µm. Using this approach, it was possible to show that below 20 µm slit width, the value of the measured FWHM begins to increase (in relation to the expected nominal one). This effect is well documented for small fields in megavoltage x‐ray beams^38^ and is due to the finite size of the x‐ray source being partially occluded by the collimator, causing an overlapping beam penumbra. If this geometrical penumbra is larger than the field size, then the FWHM of the resulting beam increases. Differences between the two vM1212 detector measurements are attributed to subtle differences when repositioning the detector and uncertainties in the reproducibility of the collimator movements, however this effect appears to be minimal.

**Figure 14:**
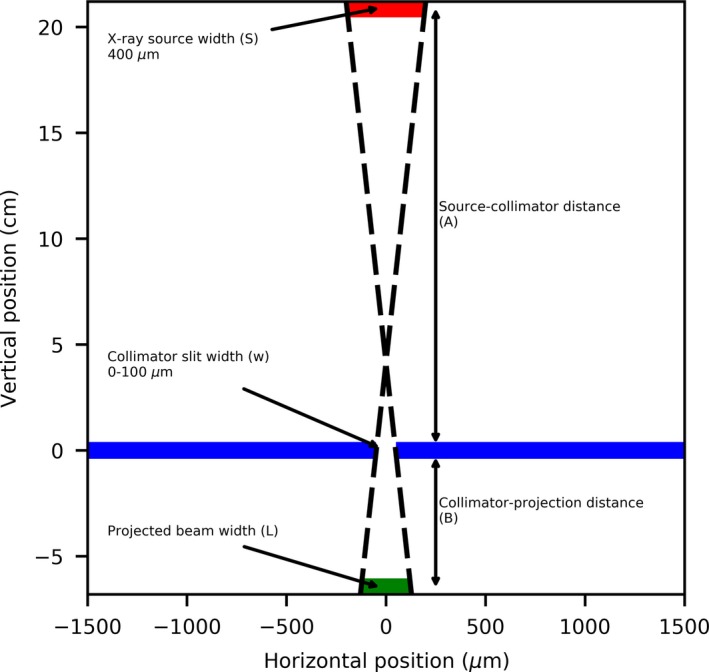
Geometric setup of the microbeam collimator, resulting in the larger full width at half maximum (FWHM). [Color figure can be viewed at http://wileyonlinelibrary.com]

The larger FWHM for all measurements can be attributed to the finite size of the x‐ray source. As shown in Fig. 14, for a finite source size (S), collimator slit width (w), source‐collimator distance (A), and collimator‐projection distance (B); the projected beam width can be approximated using Eq. (1).

(1) $$L=\left[\frac{B}{A}+1\right]w+\frac{\mathrm{BS}}{A}$$

For this approximation and to simplify the scatter effects, we assumed that the collimator is infinitely thin and consists of only one layer instead of the three that comprise the actual and previously described design of the collimator. With the previous assumptions we are considering the calculated projected beam size as an approximation of the FWHM of the microbeam peak. Using the values described previously for A, B and S, the values for the theoretical resolvable slit size were plotted on Fig. 13 for comparison with measured results. With Eq. (1), the smallest microbeam peak FWHM created by the collimator that could be possible to resolve would be equal to 128.3 ± 13.0 µm (assuming 10% uncertainty of x‐ray source size), whilst using the extrapolated results from the vM1212 detector the minimum is calculated to be 126.0 ± 0.7 µm. The differences in the slope between the derived (geometric approximation) and measured (vM1212 detector repeat linear fit) FWHMs are likely to be due to the numerous approximations and would need full Monte Carlo simulation with an accurate model of the geometry and scatter conditions.

### 3.3 Peak and Valley Measurements

**Figure 15:**
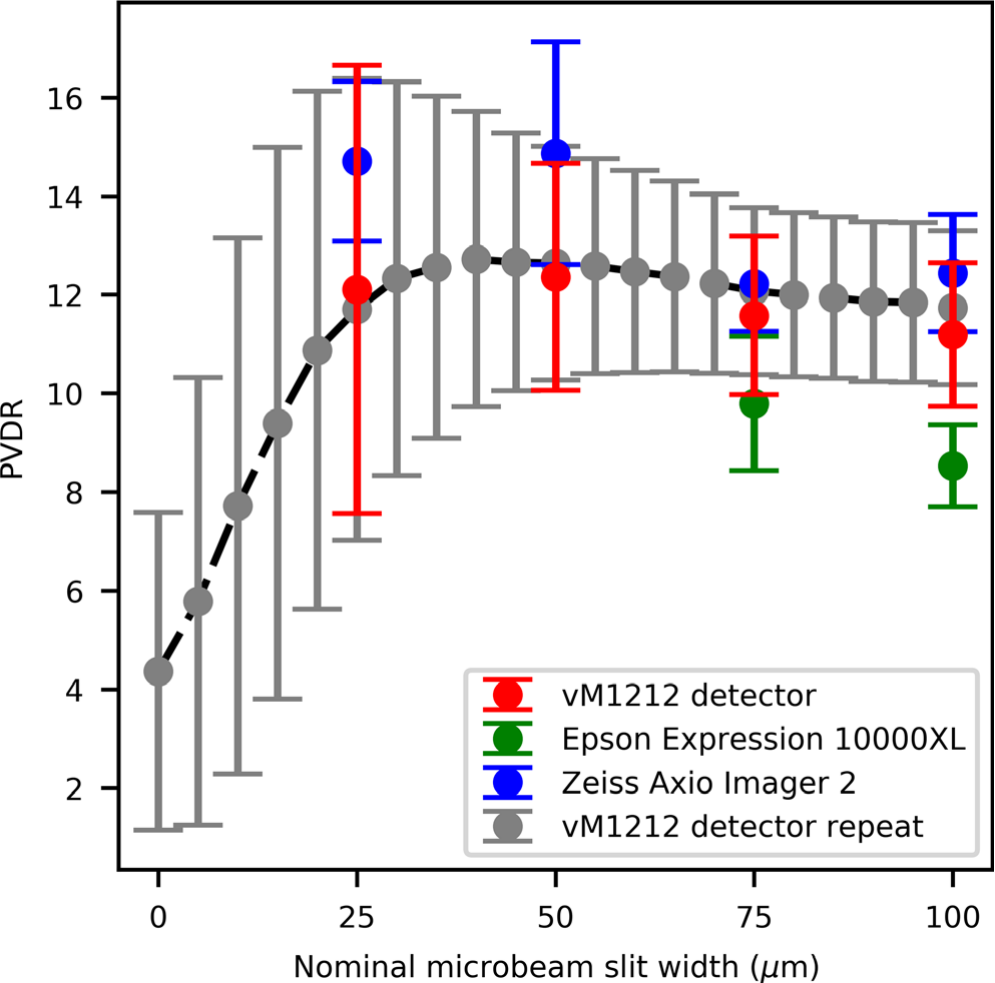
Comparison of PVDR for different slit widths. The PVDR measurements for the 25 and 50 µm slit width Epson Expression 10000XL are omitted. [Color figure can be viewed at http://wileyonlinelibrary.com]

By fitting Gaussians to each of the peaks in both the vM1212 detector and EBT3 film profiles, the Peak‐to‐Valley Dose Ratio (PVDR) can be estimated and compared to results reported in the literature (Fig. 15). The values calculated for the PVDR were comparable to what one might expect for this microbeam collimator when comparing to previous measurements in a similar collimator by Bartzsch *et al.* (where 15.5 ± 1.5 was measured at 10 mm depth),^39^ especially when considering the significantly larger SSD of this investigation.

**Figure 16:**
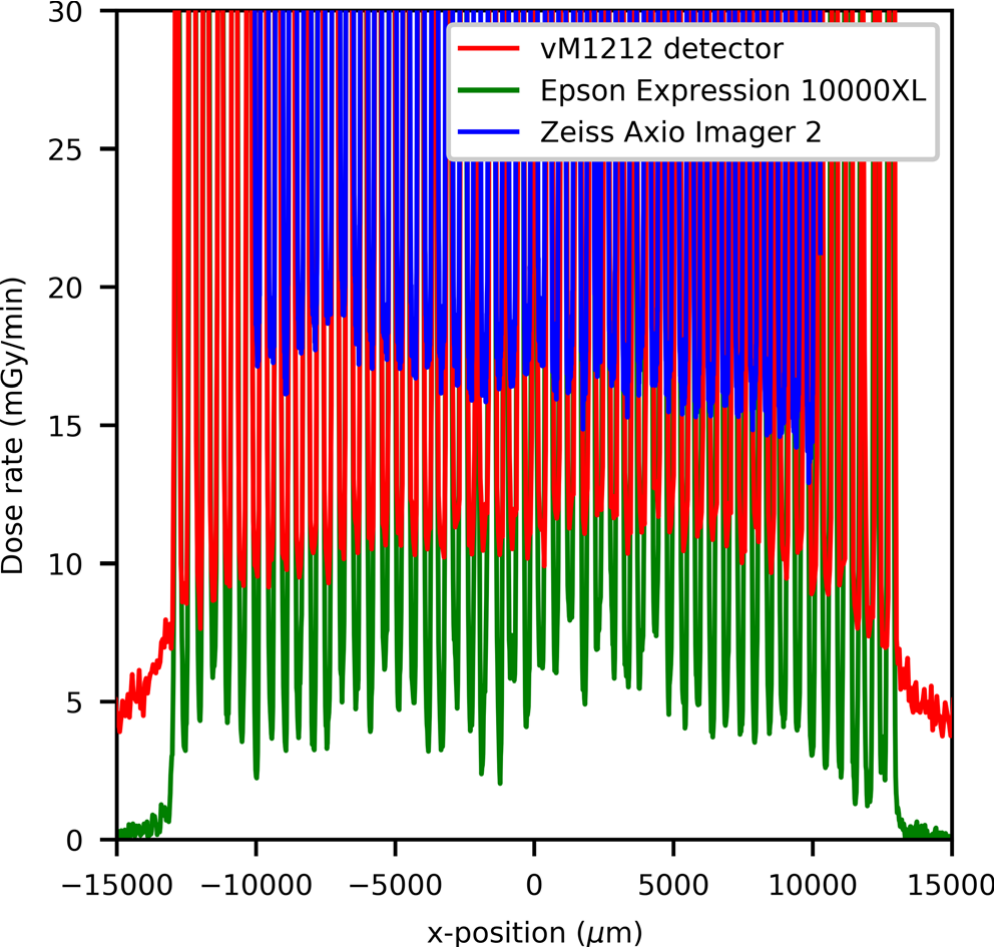
25 µm slit width valley profile comparison. [Color figure can be viewed at http://wileyonlinelibrary.com]

The PVDRs obtained using the Epson Expression 10000XL for the 25 and 50 µm slit widths were found to be significantly larger than both predicted by literature and as reported by the vM1212 detector and the Zeiss Axio Imager 2 measurements. This can be attributed to a significant under response of the Epson Expression 10000XL to the microbeam valleys, as shown in Fig. 16. It is possible that the two film method used for optical microscopy could be applied to compensate for this and record a more accurate dose profile; however this was not within the scope of the investigation.

Using the vM1212 detector it was possible to rapidly calculate the PVDR for a large number of slit widths. As shown in Fig. 9, radiation leak is present through the collimator at slit width 0 µm from which a PVDR could be calculated. The decrease in PVDR below 20 µm is consistent with the increase in FWHM as observed in Fig. 13 which was attributed to an increased proportion of the radiation resulting from scatter with decreasing slit width.

### 3.4 Modulation transfer measurements

**Figure 17:**
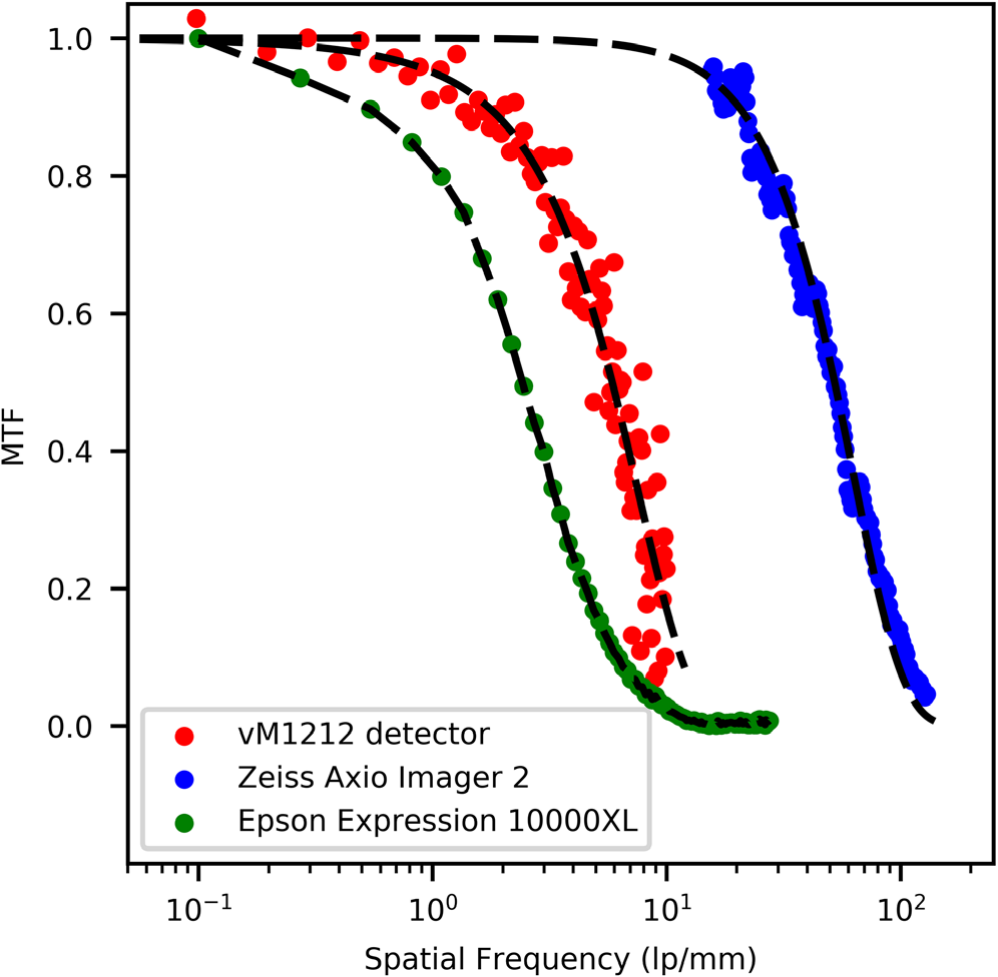
Comparison of MTF for different measurement techniques. [Color figure can be viewed at http://wileyonlinelibrary.com]

The results of modulation transfer measurements are shown in Fig. 17. It can be shown that while the spatial resolution of the vM1212 detector is better than the Epson Expression 10000XL scanner, the Zeiss Axio Imager 2 microscope is superior to both.

## 4 Discussion

In comparison to dedicated facilities such as the European Synchrotron Radiation Facility (ESRF), the x‐ray source used for this investigation was not optimized for microbeam radiotherapy with the dose rate measured after the collimator to be less than 0.05 Gy/s. This is substantially less than the dose rate used at synchrotrons for microbeam radiotherapy (often exceeding 100 Gy/s).^40^ The microbeam FWHMs delivered in this investigation are significantly larger than the 25 µm wide beams capable at the ESRF and as such, further research of the vM1212 detector under such beam conditions is required. The mean energy of this investigation (approximately 95 keV as calculated by the x‐ray emission spectra calculation software SpekCalc^41^, ^42^, ^43^) was comparable to that of dedicated synchrotrons,^44^, ^45^ however undoubtedly the effect of the different spectra must be considered.

A comparison of the three microbeam detection methods evaluated in this work can be seen in Table 2. Whilst the vM1212 detector has demonstrated the feasibility of a CMOS sensor for microbeams measurement in this investigation, significant deviations to established dosimetry methods were observed and further studies comparing to Monte Carlo simulations for relative dosimetry are still necessary. The Zeiss Axio Imager 2 remains a suitable readout method for commissioning and situations where maximum precision is required however, this method is relatively young and validated protocols and workflows need to be established to allow wider uptake for this method among microbeam community. The use of the Epson Expression 10000XL for microbeam measurements is not recommended due to the (relatively) poor spatial resolution.

**Table 2:** Comparison of the different microbeam detection methods evaluated in this work.

	Microbeam detection method		
	vM1212 detector	Zeiss Axio Imager 2 (+ EBT3 film)	Epson Expression 10000XL (+ EBT3 film)
Advantages	Real time measurement and analysis Short exposure is sufficient to obtain accurate profile information	Highest spatial resolution No dose rate dependence^49^	Lower cost Established clinical workflow No dose rate dependence^49^
Disadvantages	Limited life expectancy due to cumulative radiation damage Spatial resolution limited by 50 µm pixel pitch	24 hours self‐development Complex and time consuming analysis process	24 hours self‐development Poorest spatial resolution ad hence limited suitability for microbeam applications
	Higher price	Necessity to establish procedures and workflow for wider uptake of this method	Software licensing costs

The vM1212 detector does not possess the spatial resolution necessary for accurate microbeam dosimetry with its relatively large 50 µm pixels, compared to other quality assurance mechanisms discussed previously (such as the PTW microdiamond) with ~1 µm resolution. In addition, well established characteristics of other detection methods necessary for routine quality assurance such as dose rate and beam quality dependence have not been taken into account. The vM1212 detector operates using a "rolling shutter" frame acquisition method which does not present an issue for static or slow moving microbeam sources such as the type used in this investigation but may not be ideal for fast scanned microbeam spots. Additionally the maximum full field refresh rate of 34 fps may cause temporal blurring, however this effect could be minimized by binning pixels together or recording only a smaller region of interest. This refresh rate is still considerably lower than that of commercial radiotherapy electrometers (such as the Unidos webline with 1 kHz sampling rate^46^). Whilst the dose delivered to the films scanned by the Epson Expression 10000XL is relatively low (average peak dose of >1 Gy) for EBT3 film standards, it must be noted that the vM1212 detector is capable of obtaining similar or better quality images in less than 2 mGy per frame, highlighting its potential for real‐time microbeam verification.

Looking forward, CMOS sensors resistant to ionizing radiation have been developed for other harsh radiation environments (such as space), achieving pixel pitches of less than 10 µm^47^, ^48^ in size. The use of such sensors in the future could obtain real‐time microbeam profile information surpassing even that of the Zeiss Axio Imager 2, however making these sensors large enough to cover the same field of view as the vM1212 detector could become prohibitively expensive due the number of pixels required and sensor yield losses.

## 5 Conclusion

Microbeam radiotherapy is a rapidly developing method of cancer treatment with significant therapeutic improvements over conventional radiotherapy.^50^, ^51^ The dosimetric challenges associated with the high dose gradients in microbeam radiotherapy prevent the use of well‐established dosimetry equipment used in radiotherapy and (to date), all novel techniques for monitoring microbeams have only obtained one dimensional profile information; limiting their clinical viability.

In this study, we have demonstrated the capacity of the two dimensional vM1212 pixelated detector to discriminate individual microbeams peaks with FWHM between 130 and 190 µm. The high dynamic range of the vM1212 detector allows the signal detection of both the high dose peaks and the low dose valleys (of microbeams with less than 20 PVDR) to be measured in real‐time, which provides a significant advantage over EBT3 films requiring at least 24 hr post‐irradiation processing. Observed peak‐to‐valley dose ratios and peak to peak separations measured by the vM1212 detector were comparable those obtained using the optical microscopy method employing Zeiss Axio Imager 2 microscope. The use of pixelated sensors for in*‐vivo* beam monitoring in conventional radiotherapy beams is already being researched by multiple groups^52^, ^53^ and as the technology behind the sensors matures, it is anticipated that future CMOS detectors will have all of the required characteristics for microbeam dosimetry.

## Conflict Of Interest

Due to the prototype nature of the device, the manufacturer of the vM1212 detector, vivaMOS Ltd, has been involved in data collection providing advice and technical support throughout the investigation.

## References

[1] Slatkin DN , Spanne P , Dilmanian FA , Sandborg M . Microbeam radiation therapy. Med Phys. 1992;19:1395–400. 1461201 10.1118/1.596771

[2] Bouchet A , Serduc R , Laissue JA , Djonov V . Effects of microbeam radiation therapy on normal and tumoral blood vessels. Phys Med. 2015;31:634–41. 26004351 10.1016/j.ejmp.2015.04.014

[3] Serduc R , Christen T , Laissue J , et al. Brain tumor vessel response to synchrotron microbeam radiation therapy: a short‐term *in vivo* study. Phys Med Biol. 2008;53:3609–22. 18560052 10.1088/0031-9155/53/13/015

[4] Crosbie JC , Anderson RL , Rothkamm K , et al. Tumor cell response to synchrotron microbeam radiation therapy differs markedly from cells in normal tissues. Int J Radiat Oncol. 2010;77:886–94. 10.1016/j.ijrobp.2010.01.035 20510199

[5] Bouchet A , Serduc R , Laissue JA , Djonov V . Effects of microbeam radiation therapy on normal and tumoral blood vessels. Phys Medica. 2015;31:634–41. 10.1016/j.ejmp.2015.04.014 26004351

[6] Prise KM , Schettino G , Folkard M , Held KD . New insights on cell death from radiation exposure. Lancet Oncol. 2005;6:520–8. 15992701 10.1016/S1470-2045(05)70246-1

[7] Fernandez‐Palomo C , Bräuer‐Krisch E , Laissue J , et al. Use of synchrotron medical microbeam irradiation to investigate radiation‐induced bystander and abscopal effects in vivo. Phys Med. 2015;31:584–95. 25817634 10.1016/j.ejmp.2015.03.004

[8] Bagheri H , Soleimani A , Gharehaghaji N , et al. An overview on small‐field dosimetry in photon beam radiotherapy: developments and challenges. J Cancer Res Ther. 2017;13:175. 28643730 10.4103/0973-1482.199444

[9] Das IJ , Downes MB , Kassaee A , Tochner Z . Choice of radiation detector in dosimetry of stereotactic radiosurgery‐radiotherapy. J Radiosurg. 2000;3:177–86.

[10] Bräuer‐Krisch E , Serduc R , Siegbahn EA , et al. Effects of pulsed, spatially fractionated, microscopic synchrotron X‐ray beams on normal and tumoral brain tissue. Mutat Res – Rev Mutat Res. 2010;704:160–6. 10.1016/j.mrrev.2009.12.003 20034592

[11] Regnard P , Le Duc G , Bräuer‐Krisch E , et al. Irradiation of intracerebral 9L gliosarcoma by a single array of microplanar x‐ray beams from a synchrotron: Balance between curing and sparing. Phys Med Biol. 2008;53:861–78. 18263945 10.1088/0031-9155/53/4/003

[12] Serduc R , Bouchet A , Bräuer‐Krisch E , et al. Synchrotron microbeam radiation therapy for rat brain tumor palliation—influence of the microbeam width at constant valley dose. Phys Med Biol. 2009;54:6711–24. 19841517 10.1088/0031-9155/54/21/017

[13] Bräuer‐Krisch E , Adam J‐F , Alagoz E , et al. Medical physics aspects of the synchrotron radiation therapies: microbeam radiation therapy (MRT) and synchrotron stereotactic radiotherapy (SSRT). Phys Med. 2015;31:568–83. 26043881 10.1016/j.ejmp.2015.04.016

[14] Annabell N , Yagi N , Umetani K , Wong C , Geso M . Evaluating the peak‐to‐valley dose ratio of synchrotron microbeams using PRESAGE fluorescence. J Synchrotron Radiat; 2012;19(3):332–339. 22514166 10.1107/S0909049512005237 PMC3621279

[15] Rosenfeld AB , Kaplan GI , Kron T , et al. MOSFET dosimetry of an X‐ray microbeam. IEEE Trans Nucl Sci. 1999;46:1774–80.

[16] Kaplan GI , Rosenfeld AB , Allen BJ , Booth JT , Carolan MG , Holmes‐Siedle A . Improved spatial resolution by MOSFET dosimetry of an x‐ray microbeam. Med Phys. 2000;27:239–44. 10659763 10.1118/1.598866

[17] Livingstone J , Stevenson AW , Butler DJ , Häusermann D , Adam JF . Characterization of a synthetic single crystal diamond detector for dosimetry in spatially fractionated synchrotron x‐ray fields. Med Phys. 2016;43:4283–93. 27370143 10.1118/1.4953833

[18] PTW Freiburg . 2013 IONIZING RADIATION DETECTORS: Including Codes of Practice 100.

[19] Rosenfeld AB . Electronic dosimetry in radiation therapy. Radiat Meas. 2006;41:S134–53.

[20] Archer J , Li E , Davis J , Cameron M , Rosenfeld A , Lerch M . High spatial resolution scintillator dosimetry of synchrotron microbeams. Sci Rep. 2019;9:6873. 31053762 10.1038/s41598-019-43349-6 PMC6499773

[21] Lerch MLF , Dipuglia A , Cameron M , et al. New 3D silicon detectors for dosimetry in microbeam radiation therapy. J Phys Conf Ser. 2017;777:012009.

[22] Davis JA , Paino JR , Dipuglia A , et al. Characterisation and evaluation of a PNP strip detector for synchrotron microbeam radiation therapy *Biomed* . Phys Eng Express. 2018;4:044002.

[23] Povoli M , Alagoz E , Bravin A , et al. Thin silicon strip detectors for beam monitoring in micro‐beam radiation therapy. J Instrum. 2015;10:P11007.

[24] Bartzsch S , Lott J , Welsch K , Bräuer‐Krisch E , Oelfke U . Micrometer‐resolved film dosimetry using a microscope in microbeam radiation therapy. Med Phys. 2015;42:4069–79. 26133607 10.1118/1.4922001

[25] Anon . *GAFCHROMIC ^TM^ DOSIMETRY MEDIA*, *TYPE, EBT*‐3. http://www.gafchromic.com/documents/EBT3_Specifications.pdf

[26] Anon .Garchromic EBT Films ‐ GAFchromic^TM^ . http://www.gafchromic.com/gafchromic-film/radiotherapy-films/EBT/index.asp

[27] McLaughlin WL , Yun‐Dong C , Soares CG , Miller A , Van Dyk G , Lewis DF . Sensitometry of the response of a new radiochromic film dosimeter to gamma radiation and electron beams. Nucl Instruments Methods Phys Res Sect A: Accel Spectrometers Detect Assoc Equip. 1991;302:165–76.

[28] Li Y , Chen L , Zhu J , Liu X . The combination of the error correction methods of GAFCHROMIC EBT3 film. PLoS ONE. 2017;12:1–17. 10.1371/journal.pone.0181958 PMC5531657 28750023

[29] Tagiling N , Ab Rashid R , Azhan SNA , Dollah N , Geso M , Rahman WN . Effect of scanning parameters on dose‐response of radiochromic films irradiated with photon and electron beams. Heliyon. 2018;4:e00864. 30364574 10.1016/j.heliyon.2018.e00864 PMC6197593

[30] Sedgwick I , Das D , Guerrini N , Marsh B , Turchetta R . LASSENA: a 6.7 megapixel, 3‐sides buttable wafer‐scale CMOS sensor using a novel grid‐ addressing architecture. Proc Int Image Sens Work. 2013;3–6.

[31] Tryggestad E , Armour M , Iordachita I , Verhaegen F , Wong JW . A comprehensive system for dosimetric commissioning and Monte Carlo validation for the small animal radiation research platform. Phys Med Biol. 2009;54:5341–57. 19687532 10.1088/0031-9155/54/17/017 PMC3365538

[32] Anon . ExaFlex^TM^ ‐ MacroMedics. https://www.macromedics.com/files/upload/14/macromedics-productcatalogue.pdf

[33] Micke A , Lewis DF , Yu X . Multichannel film dosimetry with nonuniformity correction. Med Phys. 2011;38:2523–34. 21776787 10.1118/1.3576105

[34] Mayer RR , Ma F , Chen Y , et al. Enhanced dosimetry procedures and assessment for EBT2 radiochromic film. Med Phys. 2012;39:2147–55. 22482635 10.1118/1.3694100

[35] Anon . ZEISS Axio imager 2 for life science research.

[36] Anon . 2015 BSI Standards Publication Medical electrical equipment — Characteristics of digital x‐ray imaging devices Part 1–1: Determination of the detective quantum efficiency — Detectors used in.

[37] Donini B , Rivetti S , Lanconelli N , Bertolini M . Free software for performing physical analysis of systems for digital radiography and mammography. Med Phys. 2014;41:051903. 24784382 10.1118/1.4870955

[38] Das IJ , Ding GX , Ahnesjö A . Small fields: nonequilibrium radiation dosimetry. Med Phys. 2007;35:206–15. 10.1118/1.2815356 18293576

[39] Bartzsch S , Cummings C , Eismann S , Oelfke U . A preclinical microbeam facility with a conventional x‐ray tube. Med Phys. 2016;43:6301–8. 27908159 10.1118/1.4966032 PMC5965367

[40] Eling L , Bouchet A , Nemoz C , et al. Ultra high dose rate synchrotron microbeam radiation therapy. preclinical evidence in view of a clinical transfer. Radiother Oncol. 2019;139:56–61. 31307824 10.1016/j.radonc.2019.06.030

[41] Poludniowski GG , Evans PM . Calculation of x‐ray spectra emerging from an x‐ray tube. Part I. Electron penetration characteristics in x‐ray targets. Med Phys. 2007;34:2164–74. 17654919 10.1118/1.2734725

[42] Poludniowski GG . Calculation of x‐ray spectra emerging from an x‐ray tube. Part II. X‐ray production and filtration in x‐ray targets. Med Phys. 2007;34:2175–86. 17654920 10.1118/1.2734726

[43] Poludniowski G , Landry G , DeBlois F , Evans PM , Verhaegen F . *SpekCalc* : a program to calculate photon spectra from tungsten anode x‐ray tubes. Phys Med Biol. 2009;54:N433–8. 19724100 10.1088/0031-9155/54/19/N01

[44] Martínez‐Rovira I , Sempau J , Prezado Y . Development and commissioning of a Monte Carlo photon beam model for the forthcoming clinical trials in microbeam radiation therapy. Med Phys. 2012;39:119–31. 22225281 10.1118/1.3665768

[45] Livingstone J , Stevenson AW , Häusermann D , Adam J‐F . Experimental optimisation of the X‐ray energy in microbeam radiation therapy. Phys Med. 2018;45:156–61. 29472081 10.1016/j.ejmp.2017.12.017

[46] PTW . 2010 User Manual: UNIDOS webline Type 10021, Type 10022 and Type 10023.

[47] Sellier C , Gambart D , Perrot N , Garcia‐Sanchez E , Virmontois C , Mouallem W , Bardoux A . Development and qualification of a miniaturised CMOS camera for space applications (3DCM734/3DCM739) International Conference on Space Optics — ICSO 2018 vol 11180, ed Karafolas N, Sodnik Z, Cugny B (SPIE). 2019;106.

[48] Kim Woo‐Tae , Park Cheonwi , Lee Hyunkeun , Lee Ilseop , Lee Byung‐Geun . A high full well capacity CMOS image sensor for space applications. Sensors. 2019;19:1505. 10.3390/s19071505 PMC6479534 30925711

[49] Borca VC , Pasquino M , Russo G , et al. Dosimetric characterization and use of GAFCHROMIC EBT3 film for IMRT dose verification. J Appl Clin Med Phys. 2013;14:158. 10.1120/jacmp.v14i2.4111 PMC5714357 23470940

[50] Schültke E , Balosso J , Breslin T , et al. Microbeam radiation therapy — grid therapy and beyond: a clinical perspective. Br J Radiol. 2017;90:20170073 28749174 10.1259/bjr.20170073 PMC5853350

[51] Prezado Y , Jouvion G , Patriarca A , et al. Proton minibeam radiation therapy widens the therapeutic index for high‐grade gliomas. Sci Rep. 2018;8:16479. 30405188 10.1038/s41598-018-34796-8 PMC6220274

[52] Page RF , Abbott NL , Davies J , et al. Using a monolithic active pixel sensor for monitoring multileaf collimator positions in intensity modulated radiotherapy. IEEE Trans Nucl Sci. 2014;61:74–8.

[53] Bartoli A , Scaringella M , Baldi A , et al. PO‐0873: 2D pixelated diamond detector for patient QA in advanced radiotherapy treatments. Radiother Oncol. 2018;127:S459–60.

